# Apical ballooning syndrome (Takotsubo Syndrome): case report

**DOI:** 10.1186/1755-7682-6-12

**Published:** 2013-04-18

**Authors:** Charles Ulloffo do Nascimento, Carlos Eduardo da Costa Nunes Bosso, Paulo Henrique Jorge, Franciele Marques Vanderlei, Henrique Issa Artoni Ebaid, Vitor Engrácia Valenti, Luiz Carlos Marques Vanderlei

**Affiliations:** 1 Presidente Prudente Regional Hospital. R. José Bongiovani, Presidente Prudente, SP, 1297. 19050-680, Brazil; 2 Santa Casa Municipal Hospital, Heart Institution. R. Donato Armelin, 351 - Pres. Prudente, Presidente Prudente, SP, 19014-120, Brazil; 3 Department of Cardiology, Federal University of São Paulo, UNIFESP. Rua Sena Madureira, São Paulo, 1500. 04021-001, Brazil; 4 Post-graduate Program in Physical Therapy, Faculty of Sciences and Technology, UNESP. R. Roberto Simonsen, 305. 19060-900, Presidente Prudente, SP, Brazil

**Keywords:** Takotsubo cardiomyophaty, Broken-heart syndrome, Stress-induced cardiomyophaty

## Abstract

**Introduction:**

The apical ballooning syndrome (ABS) is a single reversible cardiomyopathy often triggered by a stressful event. We aimed to present a case report regarding this disorder.

**Case presentation:**

Here we present the case of a 77-year-old female hypertensive patient, sedentary and non-smoker, diagnosed with apical ballooning syndrome. We describe the clinical signs and symptoms, changes in markers of myocardial necrosis and changes in the electrocardiogram and coronary angiography.

**Conclusion:**

The course of events patient showed clinical improvement with treatment and support was not necessary to administer specific medications or interventions to reverse the situation. After hemodynamic stabilization coronary angiography showed no obstructive lesions and left ventricle with akinesia of the apex and the middle portion of the left ventricle.

## Background

The apical ballooning syndrome (ABS) or Takotsubo cardiomyopathy is a single reversible cardiomyopathy often triggered by a stressful event [1], diagnosed in 0.02% of all hospitalizations in the United States [2], however, this syndrome probably occurs due to under-diagnosed use of thrombolytics, which saves the coronary angiography intervention [3].

Chest pain and dyspnea are the typical symptoms [1]. In the laboratory tests it may be observed increase in CK, CK-MB, myoglobin and troponin. Brain natriuretic peptide (BNP) is very high (higher than 1000 pg/ml), confirming the significant dysfunction of the heart, and in 50% C-reactive protein is high (higher than 9 mg/l), which may indicate a worst prognosis [3].

In the electrocardiogram (ECG) analysis, transient elevation of the ST segment may be present and the QT interval may be longer [4]. Tachycardia is always present and 70% of patients have an arrhythmic pulse (atrial fibrillation in 6% - 7% of cases). It may be observed sinus bradycardia and atrioventricular blockade [3,5]. It is usually observed hypokinesia or akinesia in mid and apical segments of the left ventricle with preserved systolic function without obstructive coronary lesions [1]. This cardiomyopathy should be considered in patients who had sudden cardiac death without obvious heart disease [3].

Due to its unusually form of acute cardiomyopathy correctly diagnosed the ABS it is important to bring a better prognosis for the patient with the correct administration of medication. This report described a case report of ABS with emphasis on clinical signs and symptoms, changes in markers of myocardial necrosis and changes in ECG and angiography.

## Case report

**Figure 1:**
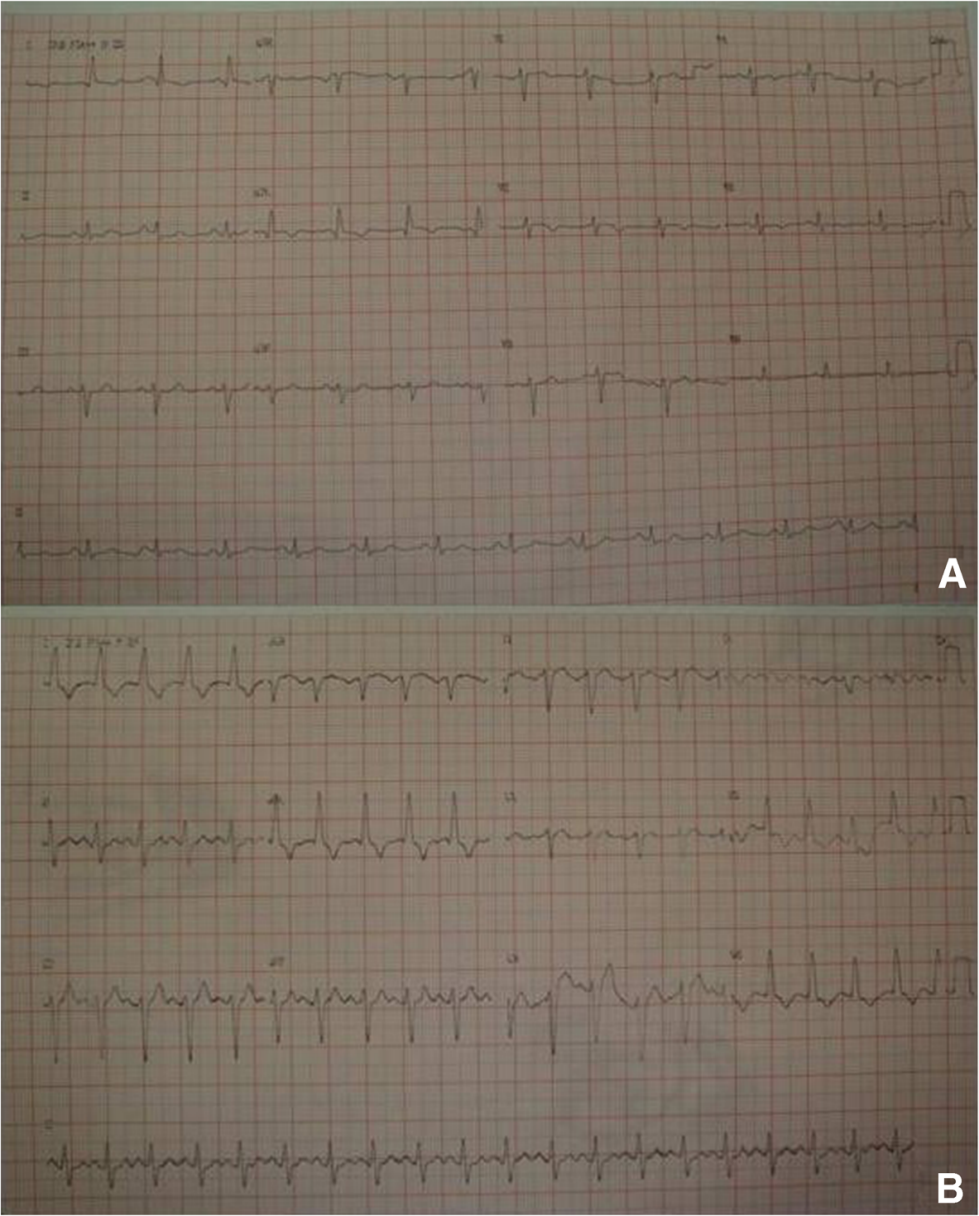
Electrocardiogram changes without (A) and left bundle branch block and left axis rotation (B).

We evaluated a female patient, 77-year-old, who was admitted in the coronary care unit (CCU) with precordial pain 5 days before the admission, after discussion with family. The patient was hypertensive, did not practice regular exercise (sedentary) and nonsmoker. Chest radiography with atheromatous plaques in the aorta (atherosclerosis). The markers of myocardial necrosis and the ECG (12-lead ECG brand DIXTAL EP-3) showed no change and the patient improved with Isordil. She was discharged in good general condition, conscious, eupneic, afebrile, anicteric and acyanotic. On physical examination she presented good auscultation and no cardiac abnormalities and laboratory tests revealed negative troponin T and CK-MB 13 ng/dl (VR: 25 ng/dl). There were no ECG changes (Figure 1A).

On the same day of discharge from the CCU the patient presented severe dyspnea, chest discomfort, paleness, cold sweats and decreased oxygen saturation, requiring supplementation. Isordil with use of discrete clinical caused brief improvement. On the electrocardiogram it was visualized left bundle branch blockade and left axis rotation (Figure 1B).

The patient returned to the CCU presenting blood pressure of 180/100 mmHg, heart rate of 113 bpm and on pulmonary auscultation we observed vesicular murmuries and diffuse wheezing. The X-ray of the chest showed prominent perihilar vascular structures, heterogeneous opacity in the right base with enhanced tendency to form interstitial reinforce, Kerley B line at the right base, grinding hemidiaphragm, loss of depth right costophrenic sinus and aorta enlarged and atheromatous. New laboratory tests showed CK-MB mass of 6.14 (VR: 3.77 ng/dl) and troponin T values of 0.3 ng/dl. After 12 hours a second sample CK-MB mass indicated value of 14.1 ng/dl.

**Figure 2:**
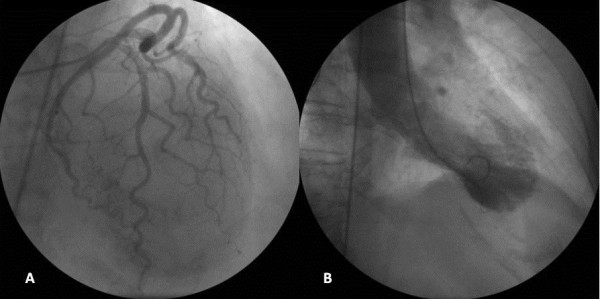
No obstructive coronary angiography (A) and ventriculography with akinesia of the apex and the middle portion of the left ventricle (B).

After hemodynamic stabilization, the patient was referred for coronary angiography that showed no obstructive lesions and left ventricle with akinesia of the apex and the middle portion of the left ventricle (Figure 2A and B). There was no recurrence of the previous case and/or hemodynamic changes and the patient was discharged to the ward in good condition.

## Discussion

This case report described the history of a patient diagnosed with ABS who obtained good clinical outcome with supportive treatment, no specific medications or interventions was necessary to reverse the syndrome. This case report may be included in the differential diagnosis of patients with acute coronary syndrome apparent changes with regional wall motion and absence of obstructive coronary pathology [1].

The ABS is reversible and triggered by physical or emotional severe stress [6], predominant in women (9:1) with a mean age between 60 and 75 years old and less than 3% of patients have less than 50 years old [1,3,4] characteristics that were observed in this case. It is possible that, in women, estrogen has a protective role on the vascular system, and a relative deficiency of estrogen after menopause may predispose to the development of ABS [1,3,4,7].

In this syndrome, the patients may present chest pain (CCS class III) in 50% to 60%, however, some of them present moderate to severe dyspnea (60%), shock or just electrocardiographic abnormalities, [4,7]. Nonspecific T wave abnormalities, new bundle branch block, and in some cases, a normal ECG can be found [1]. This report showed a normal electrocardiogram at admission, but evolved with left bundle branch block during hospitalization.

The magnitude of the increase in biomarkers was lower than that observed in acute myocardial infarction and disproportionately low to the extensive and acute abnormality regional wall motion which characterizes the ABS [1].

Most patients with this syndrome present normal coronary arteries, which was evidenced in the case described, may also present mild atherosclerosis [1].

The ABS should be included in the differential diagnosis of patients with apparent acute coronary syndrome in the left ventricle in the absence of obstructive coronary artery disease, especially in the definition of a stressful trigger [1,4]. According to Prasad et al. [1] there is a need to establish a registry to investigate ABS, its natural history and conduct of randomized trials of pharmacotherapy strategies aiming to promote myocardial recovery and prevent recurrence.

One important mechanism that is worth to be discussed as a triggering factor is the adrenergic system. Beta-adrenergic receptors are related to the GPCR family of heptahelical membrane sensors, one of the largest classes of cell-surface receptors, representing essentially the primary target of current pharmaceutical therapies. The function of beta-adrenergic receptors is regulated by several factors, such as blood catecholamines and non-catecholamine neurotransmitters and age [9]. In this context, we believe that this system is a strong candidate to be involved in ABS. Moreover, adrenergic receptors and GRK proteins (mostly GRK5 and GRK2), play a role in acute coronary syndromes [10-12]. Taken together, it is suggested that the interaction between the both systems are involved in the ABS.

Our case report present important data, since the ABS described in our study is a rare cardiac disorder poorly investigated in the literature. The ABS is a newly reported condition afflicting older women, characterized by acute left ventricular systolic dysfunction, triggered by emotionally and physically stressful events, and occurring without significant coronary obstruction [1]. Sympathetic nervous system hyperactivity has been implicated in the pathophysiology of ABS. Single nucleotide polymorphisms involving the adrenergic receptors might result in susceptibility to ABS [12]. In this context, the lifestyle may improve the sympatho-vagal balance [13] and, hence, it is indicated as an important factor to prevent the ABS.

Future studies should establish standardized criteria and guidelines for the diagnosis and clinical disease and, consequently, for their treatment and follow up [8].

## Conclusion

In summary, the early diagnosis in the ABS contributes to a better prognosis, since will be necessary interventions, minimizing further complications and, thus, optimizing the chances of survival.

## Consent

Written informed consent was obtained from the patient for publication of this report and any accompanying images.

## Competing interests

The authors declare that they have no competing interests.

## Author’s contribution

All authors participated in the revision of the manuscript. All authors determined the design, interpreted the text and drafted the manuscript. All authors read and gave final approval for the version submitted for publication.
